# How Is Chimpanzee Self-Control Influenced by Social Setting?

**DOI:** 10.6064/2012/654094

**Published:** 2012-07-12

**Authors:** Theodore A. Evans, Bonnie M. Perdue, Audrey E. Parrish, Emilie C. Menzel, Sarah F. Brosnan, Michael J. Beran

**Affiliations:** Language Research Center, Georgia State University, University Plaza, Atlanta, GA 30303, USA

## Abstract

Self-control is often required in natural situations involving interactions with other individuals, and personal self-control can be compromised if other individuals act impulsively. In this study, we tested self-control in pairs of chimpanzees in a variety of settings where at least one chimpanzee of each pair performed an established test for self-control in which candies accumulated one at time as long as the chimpanzee did not eat any of them. When tested alone, some chimpanzees exhibited greater self-control as compared to when tested alongside a chimpanzee that independently performed the same type of test. However, when the nonfocal animal freely consumed rewards while the focal chimpanzee performed the accumulation task, the self-control of some focal chimpanzees was elevated as compared to when working alone. Finally, when the focal and nonfocal animals worked jointly on the same test and the number of rewards accumulated was dependent on both animals' continued ability to inhibit eating the items, chimpanzees performed the same when housed together or in adjacent enclosures. On the whole, the effects of social setting were modest, but these results may relate to the literature on vicarious depletion of self-control, and they present interesting avenues for future research.

## 1. Introduction

Self-control, defined as choosing a better more delayed outcome over a more immediate outcome, plays an important role in many complex behaviors [1, 2]. However, surprisingly little research on self-control and delay of gratification has assessed the influence of social setting. This seems to be a major oversight, given the high impact that social situations seem to play in everyday examples of health-related behavioral regulation among humans (for a review, see [3]). Positive influences of social engagement include the use of workout partners for fitness programs and support partners in various self-help and recovery programs. Negative influences are even more prominent, especially in cases in which behavioral self-regulation is disrupted by the actions of those around the focal subject. For example, it is much more difficult to maintain the intention not to have a high calorie dessert or a cigarette after dinner when one is in the company of others who choose those behaviors than when one is alone.

There is a large body of research focused on how the social environment influences food consumption. Herman et al. [4] reviewed the three primary areas of interest: (1) social facilitation, the finding that people eat more in groups than alone; (2) social modeling, the finding that people eat a similar amount to their partner; and (3) impression management, the finding that people will modify their food intake if they believe social partners are evaluating their eating behavior. These influences may also vary as a function of the type of relationship between the individuals involved, with more matching and greater food consumption found when eating with a friend than with an unfamiliar peer [5].

In the last decade, researchers have also begun focusing on the relationship between social setting and self-regulation processes [6]. This body of research has expanded the traditional focus on intrapersonal characteristics to reveal an important role of social relationships in the self-regulatory processes of goal initiation (e.g., setting the goal to exercise regularly), operation (e.g., actually exercising), and goal monitoring (e.g., reflecting on one's exercise habits). It has even been found that empathizing with a person who is exerting self-control can deplete one's own regulation abilities [7]. For example, participants who took the perspective of an individual attempting to exert self-control in a food eating situation were later willing to spend more money in a price-depletion task and produced fewer words in a word generation task than individuals who read the same story, but were not instructed to take the other individual's perspective. These findings suggested that self-regulation can be vicariously depleted through empathy with another individual [7].

Goal regulation and eating behavior certainly involve aspects of behavior monitoring and self-control; however, we know relatively little about how social factors affect delay maintenance and self-control more broadly. Here, we tested the hypothesis that certain social aspects of delayed gratification scenarios contribute to changes in self-control. To our knowledge, self-control and delay of gratification paradigms have only occasionally been used with multiple individuals at one time to examine the social influences on delay maintenance, and we know very little about how humans or nonhuman animals (hereafter animals) would respond differently depending on the presence of other subjects in the same (or similar) situation.

In an early study in this field, Bandura and Mischel [8] examined the influence of a social model on children's self-control. Fourth- and fifth-grade children were pretested for their preference for either a small immediately available monetary reward or a larger delayed monetary reward. Four weeks later, children were tested for self-control in one of three conditions: (1) after first observing a same-sex, adult model being tested in the same scenario (live model), (2) after being told by the experimenter which selection the previously tested adult subject had made (symbolic model), or (3) after being told by the experimenter the types of rewards adult participants typically chose between (no model). In the modeled conditions, the model always represented the opposite choice option as the child had chosen in the pretest, and children's subsequent self-control choices were significantly shifted in the direction of choices represented by the models. The effect of the live model even carried over to a third test presented to these children 4-5 weeks later, further demonstrating the effectiveness of a single social demonstration in molding children's delay of gratification behavior.

In a related study, Nisan [9] presented children with the choice between an immediate food reward and a delayed, but larger food reward, and children either made this decision when alone or when in a small peer group of same-sex children. When in the group setting, the children were asked to discuss the choice among themselves before coming to a decision. For boys, the group setting facilitated delayed gratification, but this pattern was not found for girls. Nisan [9] observed that, in the majority of the boys' group decisions, the first choice that was suggested prevailed as the final decision. Thus, conformity seemed to drive the effect associated with the social setting.

More recently, McCabe and Brooks-Gunn [10] gave children two self-regulatory tasks in which they had to inhibit peeking at a toy or inhibit eating available candy for a period of time either in the presence or absence of a social partner. Children in the social condition performed more poorly than children tested in the solitary condition when asked to delay eating candy or inhibit peeking. Further, in the peeking task, children tested alone performed better across increasing ages, but performance did not increase with age in the social condition. McCabe and Brooks-Gunn [10] also reported that children in the social condition tended to imitate the behavior of those around them, whether it was peeking (impulsive response) or using strategies to avoid peeking (self-control). However, there was no relationship between children's performance in the solo conditions and their likelihood to peek at the toy or eat the treats after seeing their social partner do so. This suggests that social influences do not produce a purely quantitative change in performance by simply magnifying the level of self-control/impulsivity exhibited by children when they are alone. Instead, it suggests that being in a social situation produces a more qualitative change in the nature of the task.

To our knowledge, the only study that has tested animals in a self-control situation involving a social partner was conducted by Boysen and Berntson [11]. In this study, one chimpanzee (designated the selector) pointed to one of two different quantities of food, and then the experimenter presented the chosen quantity to a nearby second (observer) chimpanzee. The selector then received the remaining quantity. Although it was to the selector's advantage to always point to the smaller quantity in this paradigm, the chimpanzees seemed unable to inhibit responding to the larger amount, even though the selector was visibly distressed by the resulting inequitable distribution of rewards. However, once the experimenters replaced the visible foods with Arabic numerals representing the quantity of each option, the chimpanzees quickly solved the task. Boysen and Berntson [11] concluded that the chimpanzees could only overcome the predisposition to choose the larger food quantity when the symbols abstracted away the prepotency of the visible food options. This effect was later replicated with these and other chimpanzees in a test not involving a partner animal (the experimenter simply removed the chosen food option from the apparatus before delivering the nonchosen option to the chimpanzee; [12]), and so the presence of the conspecific presumably was not essential to the outcome of this test.

Given the small amount and variable nature of data from developmental and comparative studies, we examined what might happen to chimpanzee delay maintenance in social situations as opposed to conditions of testing chimpanzees alone. When tested alone, some chimpanzees are very good at waiting for accumulating rewards to grow in magnitude for delays approaching 20 minutes [13, 14], and this makes them a good model for this research question. Chimpanzees even distract themselves from attending to the accumulating rewards in ways that facilitate better self-control (e.g., by playing with toys during the delay period; [15]). However, this is the first time that two chimpanzees have been simultaneously tested in a situation where they had to maintain delay of gratification and could monitor the behavior of the partner. The partner animal played various roles in the current experiment, either working independently or jointly on the accumulation tasks or receiving free food rewards while the subject was tested. Using this partnered paradigm, we investigated the degree to which the social environment affected self-control among chimpanzees, both in terms of the mere presence of the partner as well as the partner's role in the behavioral test.

## 2. Experiment  1

The chimpanzees that we tested were accustomed to having social partners in adjacent enclosures while they performed delay of gratification (and other behavioral and cognitive) tests. However, they had never worked alongside another chimpanzee on the same type of delay of gratification test at the same time. Therefore, in the present experiment we tested chimpanzees with an accumulation delay of gratification test when they were alone as well as when they were housed adjacent to another chimpanzee working on the same type of test. Given the results of a related experiment involving children [10], we predicted that both individuals would show diminished delay maintenance despite being on independent reward delivery schedules. We also predicted that trial termination times by both chimpanzees would be correlated. In other words, seeing one partner fail to continue its own trial would negatively impact the continued delay maintenance by the other partner. If confirmed, these two effects would illustrate that such situations can negatively impact chimpanzee behavior in ways similar to those seen in humans, in which one person's impulsivity often provokes impulsivity in others.

### 2.1. Participants

We tested four chimpanzees housed at the Language Research Center (LRC) of Georgia State University. Two chimpanzees were male (Sherman, age 34; Mercury, age 20), and two were female (Lana, age 37; Panzee, age 21). The chimpanzees were socially housed with constant access to one or more conspecifics except for the purposes of behavioral testing, and all individuals voluntarily separated from group members to participate in such testing.

### 2.2. Apparatus

We used the automated item transfer device from our earlier studies of chimpanzee delay of gratification (see [14]) and this apparatus consisted of two *Med Associates* dispensers controlled by a *Dell Optiplex* personal computer via a *Keithley Instruments* digital I/O board. The dispensers released individual *M&Ms* candies into receptacles that were continually available to the participants. Each dispenser rested upon the top shelf of an AV cart and fed one of two vertically hanging rubber tubes, which we fit loosely into a capped section of slightly wider transparent tubing. The capped tubing was rested in a section of the metal mesh at the perimeter of each chimpanzee's test enclosure so that the chimpanzee could easily disconnect it from the feeder tube at any time. All chimpanzees were familiar with how to disconnect the capped tube from previous experiments (e.g., [15]).

The controlling computer program was written in *Microsoft Visual Basic* and controlled the activation of the dispensers (and therefore the delivery of food rewards). An experimenter started and stopped the program from outside of the testing area using a remote keyboard while viewing the test animals via a closed circuit monitor.

### 2.3. Procedure

To ensure that all animals were in the same state of satiation and to ensure as similar a motivational state as possible, we tested all animals first thing in the morning and had strict control over what foods they had already eaten that morning (2 carrots). Only one trial was conducted with each animal (or pair of animals) per day.

Chimpanzees were tested in two conditions: *alone* and *partner*. In the alone condition, a chimpanzee was tested in isolation (no chimpanzees were housed in immediately adjacent enclosures), and only that chimpanzee had access to a delayed reward tube. That tube was accessible in the test enclosure, and the chimpanzee could disconnect the tube and eat the contents at any time during a trial (this action ended the trial). This test is based on a manual delay of gratification task used with children [16, 17] and nonhuman animals [18–21]. All four chimpanzees had experience with both the manual and automated version of this test prior to this study [13–15, 22]. The computer program dispensed one food item every 10 seconds until the chimpanzee disconnected the collection tube (or until a maximum of 60 candies was dispensed into the chimpanzee's tube). At that point, the experimenter remotely ended the trial from outside the test area. Each chimpanzee completed 10 trials in this condition (randomly interspersed among trials in the partner condition outlined below).

In the partner condition, two chimpanzees were tested at the same time. Each chimpanzee was in its own test enclosure (adjacent to, and with full visual access of, the partner). Each chimpanzee had its own collection tube that it could disconnect at any time. The computer dispensed food items every 10 seconds, with both food items dispensed at the same time into the two collection tubes. Each chimpanzee's accumulation was independent of the actions of the partner (i.e., if one chimpanzee chose to disconnect its tube, the partner's accumulation continued as long as the partner did not disconnect its own tube). Both chimpanzees could clearly see when the partner disconnected its tube and consumed its candies. Each chimpanzee was paired with each of the three other chimpanzees in five trials, and these trials were randomly ordered and also interspersed with trials in the solo condition described above. 

For each individual, we conducted an independent samples *t*-test (two tails, alpha = .05) to compare performance in the alone and partner condition. For any individuals showing a significant difference, we further explored the pattern of responses in the partner condition. A one-way ANOVA (two tails, alpha = .05) was used to see if performance differed as a function of partner identity, and for any significant main effects, we conducted a post-hoc analysis of all possible pairwise comparisons (with Bonferroni correction, alpha = .05). 

### 2.4. Results

**Figure 1:**
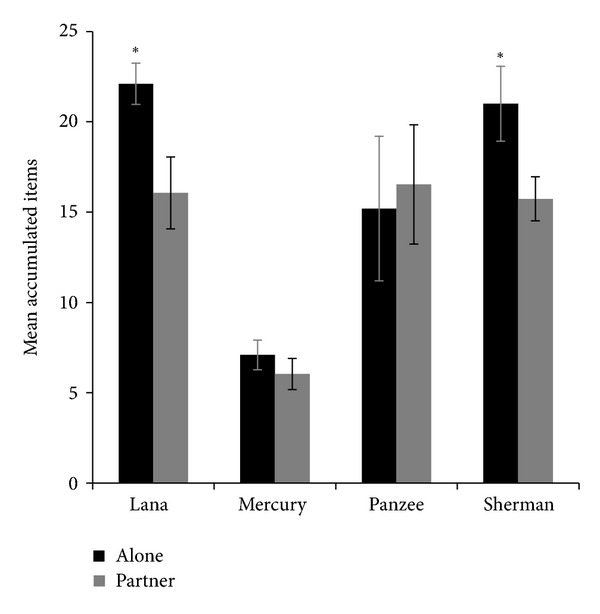
Accumulation performance as a function of the presence/absence of a conspecific working independently on the same test type in an adjacent enclosure. Error bars represent Standard Error of the Mean. An asterisk above a pair of bars represents a significant difference between conditions.

Two of the four chimpanzees showed the predicted effect whereby being tested at the same time as a partner significantly lowered the number of delayed reward items obtained (Figure 1). Lana and Sherman waited significantly longer to eat delayed rewards when they were tested alone rather than when they were tested with a partner (Lana: *t*(23) = 2.30, *P* = 0.031; Sherman: *t*(23) = 2.34, *P* = 0.028). Lana did not show a significant difference in the number of items obtained as a function of which partner she was paired with (*F*(2) = .272, *P* = 0.766). However, Sherman did obtain a different number of delayed rewards as a function of which partner he was tested with (*F*(2) = 4.382, *P* = 0.037). Post hoc comparisons indicated that Sherman obtained significantly fewer rewards when working alongside Lana in comparison to when he worked alongside Panzee (mean  difference = 7.2, *P* = 0.038), but he showed no other difference based on partner. Unlike Lana and Sherman, Mercury and Panzee showed no difference in the number of items obtained between the alone and partner conditions (Mercury: *t*(23) = 1.411, *P* = 0.172; Panzee: *t*(23) = −0.256, *P* = 0.80). They also exhibited no significant difference in the number of items obtained when working with different partners within the partner condition (Mercury: *F*(2) = .813, *P* = 0.467; Panzee: *F*(2) = .520, *P* = 0.607).

We also assessed whether there was any relation between the number of items obtained by one chimpanzee and the number of items obtained by its partner. We calculated a Pearson correlation coefficient for each focal animal for only those trials in which the nonfocal animal ended his or her trial first and the focal animal ended its trial later. There were 11 such trials for Lana, 12 for Sherman, and 7 for Panzee. Mercury was not included in this analysis because he always ended the trial before his partner. No chimpanzee showed any such relation, Lana *r*(9) = −0.035, *P* = 0.918; Panzee *r*(5) = 0.028, *P* = 0.952; Sherman *r*(10) = 0.019, *P* = 0.954.

### 2.5. Discussion

Our predictions regarding the effect of being tested alone or with a partner in a delay maintenance task were only partially supported. When chimpanzees were tested alone, two of four individuals waited for significantly longer than when tested at the same time as a partner animal (and no animals showed any facilitation in performance when tested with a partner compared to being tested alone). Further, one of the two chimpanzees that showed this effect (Sherman) seemed to be more negatively influenced by the presence of one particular partner (Lana) as compared to the others. This result was interesting given that the partner's behavior in choosing to continue to delay or choosing to end a trial had no bearing on whether the subject was allowed to continue its own trial. Thus, even though having a partner decreased the delay maintenance of two of four chimpanzees and the identity of the partner seemed to influence one chimpanzee's performance, the time at which each chimpanzee decided to end its own trial was not correlated with the time at which the partner ended its trial. Given the effect of condition (alone versus partner) on some animals' delay maintenance, one might expect some relation between the partner's actions and those of the focal animal, but this was not confirmed.

The social setting used here is not the only possible situation in which self-control or delayed gratification could be required while in the presence of other animals, and other such situations might more directly impact the subject. Thus, in the subsequent two experiments, we manipulated other aspects of the social setting and analyzed their influence on accumulation test performance. In both cases, there was always a partner animal in the vicinity, but that individual's role in the self-control scenario differed between conditions.

## 3. Experiment  2

In other social tasks, primates sometimes show interesting changes in behavior as a function of what their partner is receiving as rewards. In some species, if two primates are given the same task (e.g., exchanging tokens for individual food rewards) and one individual is receiving better rewards than the other, then the individual receiving the lesser rewards will often refuse those rewards or stop participating in the task [23, 24]. Chimpanzees are one of the species that have been reported to exhibit this aversion to inequity ([25, 26], but see [27]). These studies primarily show that aversion to inequity is focused on differences between rewards. However, one study involving capuchin monkeys indicates that, in some circumstances, primates may also be sensitive to differences in task effort [28]. Thus, it is possible that this effect may translate to a self-control situation involving two chimpanzees. We assessed this in Experiment  2 by testing one individual's ability to accumulate candies while a second individual was given free candies for the full delay interval. We expected the chimpanzees' self-control to diminish in this condition in comparison to a control condition in which a partner animal passively waited as candies accumulated in a tube outside of the enclosure and not within reach.

### 3.1. Participants and Apparatus

We tested the same four chimpanzees as in Experiment  1, but in this experiment Lana always worked alongside Mercury, and Panzee always worked alongside Sherman. As in the previous experiment, partnered individuals were tested in separate adjacent cages. The apparatus was also similar to that of Experiment  1, except that in one condition, the partner's tube was not capped and so that individual received candies freely at the same rate as the focal individual accumulated candies in the delay of gratification tube.

### 3.2. Procedure

This experiment consisted of two conditions. In one condition (hereafter referred to as the *free* condition), the partner received freely consumable candies, one at a time, at the same time and at the same rate that candies accumulated in the focal animal's capped tube (one item every 10 s) and at the same time. When each item was dispensed to the partner, it rolled down the tube and into the cage where the chimpanzee sat and could immediately pick up that item and eat it. However, once the focal chimpanzee began eating its candies, the partner chimpanzee received no further candies.

In the second condition (hereafter referred to as the *wait* condition), the partner animal's tube was placed outside of the partner's enclosure and out of reach. Once the focal animal ended the trial by beginning to eat its candies, the experimenter would end the accumulation of the partner's candies and then hand those candies to the partner. Each member of each pair was tested as the focal animal in five trials of each condition for a total of 20 trials per test pair (40 trials overall). Testing occurred approximately one year after the completion of Experiment  1.

### 3.3. Results

**Figure 2:**
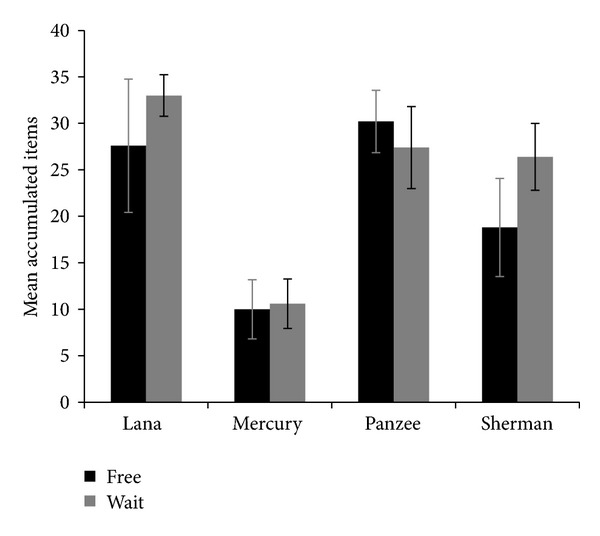
Accumulation performance as a function of whether the partner received free rewards during the delay interval (free condition) or passively waited to receive those items at the end of the test session (wait condition). Error bars represent the Standard Error of the Mean.

Figure 2 shows the mean number of items obtained by each chimpanzee in each condition (free or wait). Contrary to our predictions, chimpanzees accumulated no fewer items in the free condition as compared to the wait condition (Lana: *t*(8) = −0.719, *p* = .493; Mercury: *t*(8) = −0.145, *p* = .888; Panzee: *t*(8) = −0.372, *p* = .207; Sherman: *t*(8) = 0.439, *p* = .672). However, collapsed across condition, two chimpanzees did earn more rewards in this experiment than in the solo condition of Experiment  1 (excluding partner pairings that were not tested in Experiment  2; Lana: *t*(18) = −2.142, *p* = .046; Sherman: *t*(18) = −2.121, *p* = .048). While the other two chimpanzees also seemed to earn more rewards in Experiment  2, this difference was not significant (Mercury: *t*(18) = −1.508, *p* = .149; Panzee: *t*(18) = −1.497, *p* = .152).

### 3.4. Discussion

While the presence of a partner animal in Experiment  1 seemed to negatively affect some chimpanzees' self-control, manipulation of the equity of the self-control situation in Experiment  2 did not have a negative impact on performance. Chimpanzees accumulated a similar number of candies regardless of whether their social partner was freely consuming the same treat items throughout the delay interval or was passively waiting to receive an amount of candies that were accumulating out of their control. Further, some chimpanzees actually delayed gratification longer in this experiment than when tested alone in Experiment  1. This was contrary to our predictions, and somewhat surprising, given the presumed level of effort required to inhibit eating prepotent food rewards, and, therefore, the presumed inequity in effort required by the focal and nonfocal animals in this test. However, there are several possible explanations for this that we discuss below.

It is possible that these chimpanzees' long history of interacting with their social partners could have created a tolerance to such inequity. Brosnan and colleagues [25] have reported such an influence of social closeness on tolerance for inequitable rewards. It is also possible that, because the partner chimpanzee was not actively involved in the self-control task, the focal chimpanzee did not see this as an inequitable situation. Brosnan and colleagues [26] tested chimpanzees in a situation in which a focal animal exchanged tokens for rewards and a nonfocal animal received the same reward for doing nothing. As in the current experiment, focal chimpanzees did not exhibit any reaction to the inequity in task effort (by refusing rewards or refusing to participate more than in control conditions), regardless of whether both worked for more or less preferred rewards. 

These previous studies [25, 26] would seem to explain the results of the current experiment. However, neither of these reports indicated that task performance was enhanced (i.e., refusals were diminished) as a function of social closeness or inequitable effort in comparison to equitable situations. This was the case in the current study, in that chimpanzees accumulated more rewards in the conditions of Experiment  2 in comparison to the solo condition of Experiment  1. The difference may lie in the type of task that was involved in the present experiment (i.e., delay of gratification), and we consider this further in the general discussion.

In this experiment (as well as in Experiment  1), the social partner was always housed separately from the focal animal. This physical separation of the two chimpanzees may have caused the focal animal to ignore the partner's behavior in some conditions, since all of these individuals have had years of experience performing cognitive tests while their conspecifics were nearby and engaged in other unrelated activity. Therefore, the focus of Experiment  3 was manipulating the physical proximity of the social partner to the focal chimpanzee during the accumulation test, and making the outcome of the task contingent on the social partner. Specifically, when one animal terminated a trial, the experimenter ended accumulation for the partner as well, which was a totally novel experimental situation for these chimpanzees.

## 4. Experiment  3

In this experiment, we compared the chimpanzees' performance in two versions of a joint accumulation test in which the behavior of either chimpanzee could end the accumulation for both chimpanzees (see below). The critical difference between these conditions was whether the chimpanzees were housed separately in adjacent enclosures (as in the previous two experiments) or were housed together in the same enclosure. We hypothesized that chimpanzees would exhibit greater self-control when housed together because they would be able to interact with one another (e.g., groom, play, etc.), and this would serve as a distraction from the prepotent accumulating candies. These chimpanzees have used other things in their environment to distract themselves during a delay interval [15, 19], and we expected a social partner to serve the same purpose. We also hypothesized that social interactions would occur more frequently when the chimpanzees had to delay gratification as compared to a control condition in which candies accumulated out of their reach.

### 4.1. Participants and Apparatus

We tested the same four chimpanzees as in Experiment  1 using the same apparatus. As in Experiment  2, Lana and Mercury always worked together, and Panzee and Sherman always worked together.

### 4.2. Procedure

This experiment consisted of three conditions, and, in all three, items accumulated one at a time every 20 s for a maximum of 40 items/800 s (we increased the interitem interval from 10 s in the previous experiments to ensure that the task was sufficiently challenging, given that the chimpanzees were becoming quite practiced in variations of this test). In one condition (hereafter, the *separate *condition), the two chimpanzees were tested while housed in separate enclosures, as in Experiments  1 and 2. In the second condition (the *together-in-reach *condition), the two chimpanzees were housed together in the same enclosure and each chimpanzee had their own accumulation tube (the experimenter required each chimpanzee to sit by their respective tube before beginning the trial). In these two conditions, if either chimpanzee began eating their candies, no further candies would accumulate in *either* chimpanzee's tube, and the trial ended. In the third condition (the *together-out-of-reach *condition), the chimpanzees were again housed together, but their accumulation tubes were located outside of the enclosure and out of their reach. In these trials, the number of candies that accumulated exactly matched the number of candies the pair of chimpanzees received in the most recent together-in-reach trial. This condition was included so that we could assess whether any interactions that occurred between chimpanzees in the together-in-reach condition were simply the result of being in physical proximity or were, instead, reflective of strategic interactions designed to enhance delay maintenance. If the former case was true, chimpanzees would engage each other just as much when the tubes were out of reach, but in the latter case, interactions would be much less frequent when no delay maintenance was needed because the tubes were always out of reach.

We tested each pair of chimpanzees in 5 trials of each of these three conditions. Trial order was pseudorandomized within 3-trial blocks consisting of 1 trial of each type, with the stipulation that the together-out-of-reach trial always had to occur sometime after the together-in-reach trial. Testing occurred approximately two years after completing Experiment  2. Two experimenters (authors ECM and TAE) independently coded the chimpanzees' behavior from video after the experiment was completed. The experimenters coded the frequency and duration of all social interactions between partnered chimpanzees as well as instances in which a chimpanzee moved further than an arm's length away from his or her accumulation tube. There was substantial agreement between the two observers for both the frequency and duration of these behaviors (100% and 92.32% agreement, resp.).

### 4.3. Results

**Figure 3:**
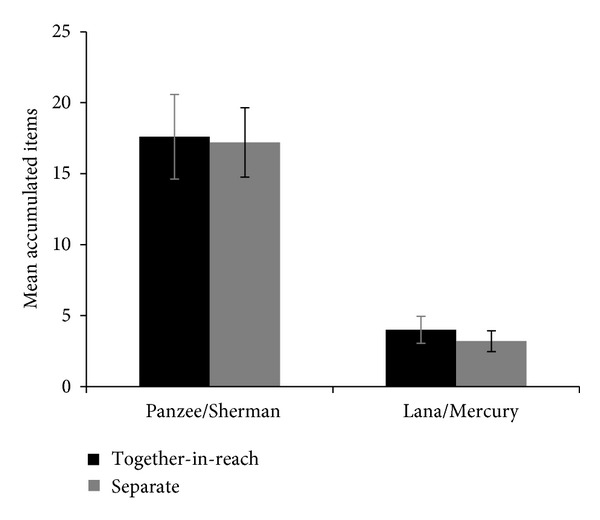
Chimpanzee performance in a joint accumulation test as a function of whether the partner animals were housed together or in separate adjacent enclosures. In both cases, chimpanzees' accumulation tubes were within reach (items accumulated in the together-out-of-reach condition was out of the chimpanzees' control). Error bars represent the Standard Error of the Mean.

Figure 3 shows the mean number of items obtained by each chimpanzee in each condition in which they had access to the accumulation tubes (separate and together-in-reach). Neither pair of chimpanzees exhibited a difference in performance between conditions (Lana/Mercury: *t*(8) = −0.667, *p* = .524; Panzee/Sherman: *t*(8) = −0.106, *p* = .918). Within each pair, one chimpanzee tended to take his tube first in most trials (Mercury: 8/10 sessions; Sherman: 9/10 sessions).

Both pairs of chimpanzees exhibited few social interactions in either condition in which they were housed in the same enclosure (Sherman once initiated a14s play bout with Panzee in a together-in-reach session; Mercury once initiated an interaction with Lana by clapping his hands together, and the pair then alternated clapping for 11-s during a together-out-of-reach session; Mercury twice initiated interactions with Lana by briefly (2 s and 5 s) flicking water at her from the floor during two together-out-of-reach sessions). 

Interestingly, when chimpanzees had physical access to their accumulation tube and were housed in the same enclosure as their partner, they were still occasionally willing to walk away from their collection tube and move about the test enclosure (Lana: *n* = 1, duration = 15 s; Mercury: *n* = 4; mean  duration = 45 s; Panzee: *n* = 0; Sherman: *n* = 6; mean duration = 101 s). In only one of these occasions did the partner chimpanzee (Lana) take the focal chimpanzee's (Mercury's) accumulation of candies after he had walked away.

### 4.4. Discussion

In this experiment, we found no influence of partner proximity/housing on chimpanzees' self-control. They were equally likely to delay gratification (or stop delaying gratification) whether they did or did not have physical access to their partner. Further, little social behavior was seen when the chimpanzees had physical access to one another, irrespective of condition. Thus, they did not take advantage of the potentially distracting nature of such interactions that possibly could have resulted in longer delay of gratification (and more accumulated rewards). It may have been possible that chimpanzees refrained from interacting with each other because they were told by the experimenter to sit by their tube at the beginning of the trial. However, this verbal cue was not necessary on all trials, as chimpanzees sometimes each sat by a different tube as the experimenter set up the task. It is also possible that chimpanzees did not interact much because they did not want to leave their tubes unattended for fear of losing their candies to their partner. Yet, neither of these potential explanations can fully explain the chimpanzees' pattern of results because chimpanzees did occasionally walk away from their tubes during sessions in which both animals' had physical access to their accumulation tubes, and this seldom led to the loss of their candies (and the one instance this happened did not prevent the focal animal from walking away from his accumulation tube in future sessions).

## 5. General Discussion

In this series of experiments, chimpanzees were mostly successful in delaying gratification to obtain multiple reward items across a variety of social conditions, and these conditions had limited influence on the overall number of rewards chimpanzees were able to accumulate by delaying gratification. Two of four chimpanzees delayed gratification for longer when tested alone than when in the presence of a social partner; however, the point at which they ended their accumulation trials did not relate to when their partners ended their trials. Additionally, when tested in the presence of a partner that received freely consumable rewards, chimpanzees showed no detriment in self-control as compared to when that partner was present but did not have access to its rewards until after the focal animal ended the trial. Finally, even when two chimpanzees delayed gratification in the same enclosure as one another and the accumulation of rewards was dependent upon both animals inhibiting eating their rewards, these chimpanzees' self-control did not diminish in comparison to control trials. Overall, chimpanzee self-control was only slightly affected by the presence or behavior of a social partner in the current task.

It is interesting to compare these results with what is known about human self-control in social situations. Of the few reports that tested social influences on self-control, the study conducted by McCabe and Brooks-Gunn [10] with 3-to-5-year-old human children most closely matches the current study in terms of social setting. In tests involving delaying eating a snack item and inhibiting peeking at a gift, children exhibited decreased self-control when tested in peer groups in comparison to when tested alone. Also, in that study, there was no clear relationship between the behavior of an individual and the behavior of their peers. Imitation of rule-breaking behavior (i.e., eating a candy too early or peeking while an experimenter wrapped a gift) did not seem to explain the deficit in performance exhibited by children tested in the peer group. Thus, both human children and (some) chimpanzees appear to struggle with delay of gratification more in a social setting than when tested alone, yet neither show a clear influence of the performance of conspecifics on their own performance.

If chimpanzees show differences in self-control simply on the basis of having a partner working at the same time on an independent schedule, as compared to being tested alone, then why were there not further differences in performance between the different social settings presented in the subsequent experiments? It is possible that the mere presence of a social partner is the only variable that matters to chimpanzees in this kind of task and, therefore, the behavior of the partner during that task had no effect on their performance. However, the chimpanzees' performance in Experiment  2 did differ from the solo condition of Experiment  1, and so this explanation seems unlikely. Moreover, chimpanzees' self-control in Experiment  2 was higher than that seen in the solo condition of Experiment  1 (whereas the opposite relationship was seen between the social and solo conditions of Experiment  1), and this points to the likelihood that the social settings used in at least those two experiments were qualitatively different for the chimpanzees. Further, although we cannot directly compare the results of Experiment  3 with the previous experiments (due to the difference in inter-item interval), it is interesting to note that the chimpanzees' performance in the last experiment resembled that of the Experiment  1 social condition considerably more than that of Experiment  2. Thus, after some chimpanzees exhibited elevated performance in Experiment  2, their performance dropped in Experiment  3, and this may point to an important difference between the social setting of Experiment  2 and the social settings of the other two experiments.

Taken altogether, these results suggest that some chimpanzees' ability to delay gratification is lessened when they observe a conspecific engaged in the same task as them, but their delay of gratification is improved when they observe a conspecific eating rewards while not participating in the task. This may suggest that some chimpanzees experience something akin to the vicarious depletion of self-control that is reported in the human literature. This is the interesting idea that self-control may operate analogously to muscles, with decreased ability and efficiency as a result of sustained exertion [29, 30] and that this decrease in performance may occur more rapidly when observing or imagining self-control depletion by another individual ([7] see also [31]). With regard to the current study, when seeing a partner animal exerting self-control, chimpanzees' own self-control may possibly be depleted at a faster rate than when they are delaying gratification on their own in an otherwise comparable situation. That said, further studies specifically designed to test this concept in chimpanzees and other animals will need to be conducted to more formally pose this possibility.

Given that these experiments were conducted with only 4 chimpanzees, additional animals should be tested to assess the validity of the modest results reported here. These chimpanzees had years of experience in delay of gratification tests, and this may have reduced the impact that the social settings tested here could have had on their performance. Considerably less experienced animals may prove to be less focused on the task of maximizing rewards and, therefore, may be more susceptible to the potential influence of a social partner's presence and/or behavior. Moreover, very little is known about how social settings and social interactions may influence self-control and self-regulation in animals (and humans) more generally. Therefore, further studies should be conducted to test other possible scenarios than those considered here. These avenues for research should provide a fuller account for the self-control capacities of animals and humans and offer insight into the emergence of behavioral self-regulation.
